# Psychometric Properties of Part 1 of the Persian Relative Mastery Scale in Stroke Survivors

**DOI:** 10.1155/oti/4334160

**Published:** 2026-11-15

**Authors:** Mahla Motahari, Akram Azad, Amin Ghaffari, Laleh Lajevardi, Ghorban Taghizadeh

**Affiliations:** ^1^ Department of Rehabilitation Sciences School of Allied Medical Sciences Mazandaran University of Medical Sciences Sari Iran mazums.ac.ir; ^2^ Rehabilitation Research Center Department of Occupational Therapy School of Rehabilitation Sciences Iran University of Medical Sciences (IUMS) Tehran Iran iums.ac.ir; ^3^ Department of Occupational Therapy Musculoskeletal Research Center School of Rehabilitation Sciences Isfahan University of Medical Sciences Isfahan Iran mui.ac.ir; ^4^ Department of Occupational Therapy Isfahan Neurosciences Research Center School of Rehabilitation Sciences Isfahan University of Medical Sciences Isfahan Iran mui.ac.ir; ^5^ Geriatric Mental Health Research Center Department of Occupational Therapy School of Rehabilitation Sciences Iran University of Medical Sciences (IUMS) Tehran Iran iums.ac.ir

**Keywords:** occupational adaptation, Relative Mastery Scale, reliability, stroke, validity

## Abstract

**Purpose:**

The Relative Mastery Scale (RMS) is a patient‐reported outcome measure that evaluates three key features of occupational adaptation: effectiveness, efficiency, and satisfaction. This study is aimed at investigating the psychometric properties of Part 1 (the six‐item quantitative component) of the RMS in stroke survivors.

**Materials and Methods:**

A total of 100 stroke survivors were recruited using a convenience sampling method. Participants were evaluated using the RMS, Canadian Occupational Performance Measure, Satisfaction With Life Scale, and other validated tools construct validity (hypothesis testing) and known‐groups validity. To investigate the test–retest reliability, 76 participants were reassessed by RMS after 2 weeks. Content validity, construct validity (hypothesis testing), known‐groups validity, structural validity, test–retest reliability, internal consistency, minimum detectable change, and floor and ceiling effects were examined.

**Results:**

The RMS showed satisfactory face and content validity. Construct validity (hypothesis testing) of the RMS was supported by significant correlations with the Canadian Occupational Performance Measure, Satisfaction With Life Scale, and other comparator instruments (*r* = −0.40–0.73). Known‐groups validity analysis indicated that the RMS could differentiate between individuals with varying levels of disability as measured by the modified Rankin Scale (*p* < 0.001). Exploratory factor analysis identified one factor. The internal consistency was excellent (*α* = 0.89; *ω* = 0.90). The test–retest reliability, assessed using the intraclass correlation coefficient (ICC), was very high (ICC = 0.98). Item‐level agreement was high (quadratic‐weighted kappa 0.91–0.99). Absolute reliability (standard error of measurement) was 0.638 (95% CI: 0.55–0.88), and the minimum detectable change was 1.76 (95% CI: 1.53–2.44). No floor or ceiling effects were detected for the total score of the Persian RMS.

**Conclusions:**

The findings suggest that Part 1 of the Persian version of the RMS is valid and reliable for assessing relative mastery in stroke survivors.

## 1. Introduction

Stroke ranks as the second leading cause of death and the third primary cause of disability globally [1]. Nearly two‐thirds of stroke survivors live with ongoing disability, and many live with unmet needs, including long‐term occupational problems [2]. Strokes have different effects on each person and can cause complicated neurological impairments in motor, sensory, cognitive, or emotional functions [3]. These complications may result in long‐term disability and participation restrictions among stroke survivors. The World Health Organization classifies disability into three categories: impairment of body function, limitations in activity, and restrictions in participation, all of which are impacted by stroke [4]. People who have had a stroke may encounter physical and psychological challenges as a result of role loss, higher caregiving responsibilities, and a lower quality of life caused by poor performance and occupational adaptation [5].

Occupational therapy interventions in people with stroke is aimed at improving the ability to perform activities of daily living by reducing activity limitations and educating people to achieve the highest possible level of autonomy, thereby improving their well‐being and quality of life. Therefore, an occupational therapist plays an important role in the rehabilitation of stroke survivors by helping stroke survivors maximize their existing abilities and re‐engage in meaningful daily activities [6–8]. One of the key approaches to achieving this goal is the occupational adaptation model (OAM), which emphasizes the individual′s ability to adapt to environmental demands and challenges in their daily activities. This approach not only enhances functional recovery but also promotes long‐term independence by focusing on the patient′s internal adaptive capacity [9].

The “OAM” [10] defines occupational adaptation as the process by which an individual and their environment address a challenge in an occupation, reflecting their response to that challenge [10, 11]. There are three primary elements in this model: the person, the occupational environment, and the drive for mastery, which function as interactive processes [9].

The concept of relative mastery, which represents the outcome of intervention within the OAM, refers to an individual′s evaluation of their occupational responses to contextual demands in terms of effectiveness, efficiency, and satisfaction [9, 10, 12].

The previously described elements are included in the “Relative Mastery Scale” (RMS) created by George‐Paschal et al. The RMS, as a means of self‐assessment, especially when current interventions prove ineffective, inefficient, or unsatisfactory, may help clients identify the need for adaptation or the development of new solutions to occupational problems. The RMS is a brief self‐report instrument that enables individuals to evaluate their occupational responses in terms of effectiveness, efficiency, and satisfaction. By allowing clients to select meaningful activities and reflect on their own performance, the instrument may facilitate occupational adaptation and support client‐centered rehabilitation [13]. This tool was translated into Persian by Hassani Mehraban et al., who also investigated its psychometric properties in 150 caregivers of patients with multiple sclerosis (MS) and found acceptable reliability and validity for evaluating mastery in Persian‐speaking caregivers [14]. Previous studies have investigated the psychometric properties of the RMS in healthy adults and MS caregivers [13, 14].

Although the RMS has demonstrated satisfactory validity and reliability in healthy adults and caregivers of patients with MS, these findings cannot be directly generalized to stroke survivors. Stroke survivors exhibit distinct cognitive, motor, and emotional characteristics that differ substantially from those of healthy individuals and caregivers of patients with MS [15]. These differences may influence the factor structure, construct validity, and internal consistency of the scale [16]. To our knowledge, no previous study has examined the psychometric properties of the Persian RMS in stroke survivors. Establishing the measurement properties of the scale in this population is essential before its use in clinical and research settings. Therefore, the present study is aimed at evaluating the psychometric properties of Part 1 of the Persian version of the RMS, consisting of six quantitative items, in Iranian stroke survivors. We hypothesized that the Persian RMS would demonstrate acceptable structural validity, construct validity, internal consistency, and test–retest reliability in stroke survivors.

## 2. Methods

### 2.1. Study Design and Participants

In this cross‐sectional, descriptive, analytical study, 100 people with stroke were selected using a convenience nonprobability sampling method, from patients referred to treatment and rehabilitation centers, between January and June 2024. The sample size was determined based on recommendations for validation studies, which generally suggest 2–20 participants per item [17], whereas some methodological guidelines indicate that approximately three to five, or up to 10, participants per item are sufficient for validity and reliability analyses [18]. Accordingly, the patient‐to‐item ratio in this study was 16.67, which falls within the recommended range for validation studies. However, it should be noted that a formal a priori power analysis was not performed to determine the sample size. A post hoc sensitivity analysis indicated that with a sample of *N* = 100, the study had 80% power at *α* = 0.05 to detect correlations as low as *r* ≈ 0.28 for construct validity testing and with *n* = 76 for the test–retest subsample, to estimate an ICC of 0.70 with reasonable precision. These values align with the psychometric endpoints of interest in this study; nonetheless, we acknowledge this post hoc approach as a limitation relative to a formal a priori sample size calculation. Of the 100 participants enrolled, 76 completed the retest assessment approximately 2 weeks later; the remaining 24 were lost to follow‐up, primarily due to being unreachable or unavailable for the second assessment within the study timeframe. Baseline characteristics of completers and noncompleters are compared in Section 3.3. Only individuals who met the eligibility criteria for the study were included. The inclusion criteria were as follows: a first‐time stroke based on the neurologist′s diagnosis; a minimum of 6 months since the stroke; acceptable cognitive function instructions (Montreal Cognitive Assessment ≥ 21 that permission has been granted for its use) [19, 20]; no other neurological, psychological, or orthopedic conditions; and the ability to communicate in Persian. Participants were excluded from the study if they experienced a recurrent stroke during the study, failed to cooperate during testing, or were diagnosed with severe depression (i.e. Hospital Anxiety and Depression Scale‐depression subscale ≥ 11) [21, 22]. This study was approved by the Ethics Committee of Iran University of Medical Sciences (Approval Code: IR.IUMS.REC.1402.681; Approval Date: November 14, 2023). Written informed consent was obtained from all participants. An experienced occupational therapist assessed all participants using the RMS, Canadian Occupational Performance Measure (COPM), Satisfaction With Life Scale (SWLS), Stroke Impact Scale‐16 (SIS‐16), Positive and Negative Affect Schedule (PANAS), and modified Rankin Scale (mRS). The assessments were conducted randomly. To avoid fatigue, rest intervals were provided between the completion of each questionnaire. After 2 weeks, 76 participants were reassessed with the RMS to assess its test–retest reliability.

### 2.2. Procedures

The Persian version of the RMS used in the present study was adopted from the version translated and culturally adapted by Hassani Mehraban et al., who followed the standardized IQOLA protocol for crosscultural adaptation. In our study, we did not conduct any new translation or linguistic modification of the RMS items. Instead, we employed the existing validated Persian version by Hassani Mehraban et al. without any changes to item wording or structure. However, considering that the psychometric validation by Hassani Mehraban et al. was conducted on a different population (caregivers of patients with MS) and that tool validity may vary across clinical groups, we reassessed face and content validity of the Persian RMS specifically in stroke survivors. This step is aimed at ensuring the comprehensibility and contextual relevance of the items in this new target population while retaining the original item structure. Formal cognitive debriefing interviews place considerable verbal and executive demands on participants and are difficult to standardize in a stroke population, where mild cognitive difficulties remain common even among individuals meeting standard cognitive inclusion criteria (MoCA ≥ 21). We therefore used a standard expert‐based approach to reassess face and content validity, consistent with established psychometric methodology [23].

After obtaining authorization from Doctor Lorrie George‐Paschal to use the RMS, the validity and reliability of the scale were assessed according to the COSMIN taxonomy of measurement. To evaluate validity, face validity, content validity, construct validity (hypothesis testing), known‐groups validity, and structural validity were examined. For reliability assessment, internal consistency, test–retest reliability, and both relative and absolute repeatability were considered. Additionally, the minimum detectable change (MDC), as well as ceiling and floor effects, were examined in stroke survivors.

### 2.3. Validity

Quantitative face validity was computed using the impact score (IS). The content validity of the RMS was evaluated using the content validity index (CVI) and the content validity ratio (CVR). The evaluation of validity in this study was guided by the COSMIN taxonomy of measurement properties, encompassing content validity, structural validity, construct validity (hypotheses testing, including known‐groups validity), internal consistency, reliability, and measurement error. Construct validity (hypotheses testing) in this study was based on the OAM, originally proposed by Schkade and Schultz, which conceptualizes relative mastery as a dynamic outcome of successful adaptation to occupational challenges. Relative mastery reflects an individual′s subjective sense of effectiveness (goal attainment), efficiency (resource use), and satisfaction (internal and external approval) in meeting occupational demands [10]. This multidimensional construct sits at the intersection of performance, emotional regulation, and quality of life [9, 10]. Because the RMS is a new scale with limited psychometric research, selecting comparator instruments to assess its construct validity (hypotheses testing) was methodologically challenging. Therefore, the COPM, SWLS, SIS‐16, and PANAS were selected, each described below.

Based on the conceptual proximity of each comparator instrument to the effectiveness, efficiency, and satisfaction dimensions of the RMS, we hypothesized positive correlations between the RMS and COPM‐performance, COPM‐satisfaction, SWLS, and SIS‐16, given their direct conceptual overlap with occupational performance, satisfaction, and functional adaptation. We hypothesized a comparatively weaker positive correlation between the RMS and PANAS positive affect and a comparatively weaker negative correlation with PANAS negative affect, given the more distal relationship between general affect regulation and occupational mastery.

The *COPM* was selected because it assesses both performance and satisfaction with daily activities, providing a direct operational match with the effectiveness and satisfaction dimensions of the RMS. The COPM emphasizes client‐centered evaluation of occupational performance, making it theoretically congruent with the adaptive function of mastery in the OAM framework [14, 24, 25].

The *SWLS* captures the cognitive appraisal of one′s overall life satisfaction, which corresponds to the satisfaction dimension of relative mastery. Higher levels of perceived mastery should intuitively align with greater life satisfaction, especially in populations coping with functional limitations [10, 12, 26].

The *SIS-16* evaluates the physical functioning of stroke survivors and thus reflects the effectiveness and functional adaptation aspects of mastery. In line with the OAM, physical functioning can be considered a prerequisite or corollary to adaptive success [9, 10, 27].

The *PANAS* provides insight into affective regulation, which has been linked to adaptive capacity and perceived success in occupational engagement [28]. Emotional responses to occupational challenges can enhance or hinder mastery, making affective balance a relevant construct for construct validity testing [29].

Although no formal structural model guided these selections, the instruments were chosen based on their construct proximity to the mastery components defined by the OAM and their widespread use in rehabilitation research. As such, these tools offer a practical and theoretically consistent set of comparators for assessing the construct validity (hypotheses testing) of the Persian RMS in a stroke population.

Known‐groups validity was investigated by comparing individuals with different levels of disability as measured by the mRS, as well as by comparing different age groups. Structural validity was assessed through an exploratory factor analysis (EFA).

### 2.4. Reliability

To assess the internal consistency of RMS, Cronbach′s alpha was used to calculate the first‐order evaluation scores. Weighted Cohen′s kappa determined the test–retest reliability of each item. Intraclass correlation coefficient (ICC) was used to check relative repeatability, whereas the standard error of measurement (SEM) assessed absolute repeatability. The MDC and the ceiling and floor effects of this test were investigated in stroke survivors.

### 2.5. Measurements

RMS was designed based on the definitions presented in the OAM and evaluates individual′s perceptions of their effectiveness, efficiency, and satisfaction with their responses to occupational challenges. The RMS consists of two parts: The first part contains six statements that are rated on a 5‐point scale (ranging from −2 to +2), where −2 indicates the lowest and +2 indicates the highest degree of relative mastery. The second part consists of three open‐ended questions related to the individual′s performance in a chosen occupational activity. The higher a person′s total score on this scale, the greater their relative mastery [13, 14]. This study focused on Part 1 of the RMS, which aligned with its psychometric aims. Part 2, containing open‐ended items, was not included as it required a qualitative approach beyond the study′s scope.

COPM is a semistructured interview in which the therapist helps the client to identify and prioritize five problems in performing daily activities and then rate their current performance and satisfaction using a 10‐point scale. COPM helps occupational therapists determine changes in the client′s occupational performance in daily life over time. The results obtained from the studies show that the Persian version of the COPM has adequate validity and reliability [25, 30].

SWLS is a self‐report scale comprising five items that measure the cognitive component of subjective well‐being. It can be easily used in questionnaires and requires only 1 minute of the respondent′s time. Each item is scored on a Likert scale from 1 (*strongly disagree*) to 7 (*strongly agree*). The total score on the scale can range from 5 to 35, with higher scores indicating greater life satisfaction. According to evidence, the validity and reliability of the Persian version of the SWLS were confirmed [26, 31].

PANAS is a 20‐item self‐report measure that includes two 10‐item mood scales: one for positive affect and one for negative affect. It was created to evaluate these two broad areas of affect. Each item on this scale is rated by the respondent on a 5‐point Likert scale, ranging from 1 (*very low*) to 5 (*very high*). The range of scores for each subscale is 10–50. The Persian version of the PANAS had good psychometric properties, and it can be used for research and evaluation of positive and negative affect in Iranian adolescents [32, 33].

SIS‐16 is one of the most reliable tools for examining the effects of stroke. Duncan et al. published SIS‐16 which is the physical function subscale of the SIS. Patients are asked to rate how much difficulty they have had in the past 2 weeks in completing each item. The measure utilizes a 5‐point Likert scale that ranges from 1 (*extremely difficult*) to 5 (*not difficult at all*). The higher score indicates a better physical function. The results of the study revealed that the Persian version of the SIS is a psychometrically appropriate instrument to assess the people with stroke. The translated Iranian version of the SIS‐16 was used in the present study and was proven to be acceptable and valid [27, 34].

*mRS* is the most practical measure of the global functional health index, emphasizing the severity and degree of physical disability and independence of a person with a stroke. This rating scale classifies people with a stroke into one of seven general disability levels, ranging from 0 to 6: 0 = *no symptoms*; 1 = *no significant disability despite some symptoms*; 2 = *slight disability*; 3 = *moderate disability, requiring some assistance but able to walk independently*; 4 = *moderately severe disability, unable to walk without assistance*; 5 = *severe disability, requiring constant care*; and 6 = *death* [35, 36]. In the present sample, participants′ mRS scores ranged from 0 to 5; no participant had an mRS score of 6, as this category corresponds to death and is inapplicable to a living stroke survivor cohort.

Demographic questionnaire included questions about age, sex, education level, marital status, employment status, dominant hand, affected side, comorbidities, medications, and using assistive devices. Clinical data such as stroke type and time since the stroke were obtained from the medical records of the participants.

### 2.6. Statistical Analysis

Normal distribution of data was confirmed by the Kolmogorov–Smirnov test. To determine face and content validity, the scale was emailed to 10 occupational therapy specialists with at least 5 years of experience in stroke rehabilitation. IS was used to calculate quantitative face validity, according to the formula IS = Importance × Frequency (*%*). The importance of each question in the questionnaire was determined on a 5‐point Likert scale from 1 (*not at all important*) to 5 (*completely important*), and frequency represents the percentage of experts rating the item as important. If the IS > 1.5, that question is considered acceptable [37]. For content validity, the CVR was used to determine the necessity of the items, calculated according to the formula CVR = (*N* *e* − *N*/2)/(*N*/2), where *N* *e* is the number of experts rating an item as “essential” and *N* is the total number of experts. According to the Lawshe table, if the number of experts is 10, CVR > 0.62 is acceptable. The CVI was used to determine the clarity of the items; to accept each item′s clarity, CVI > 0.79 is considered appropriate [23]. Pearson′s correlation coefficient was used to evaluate construct validity (hypothesis testing). A Pearson ≥ 0.90, 0.70–0.89, 0.50–0.69, and 0.26–0.49 indicate very strong, strong, moderate, and weak correlations [38]. To investigate known‐groups validity, people with stroke were divided into four clinically meaningful groups based on the mRS: no symptoms or no significant disability (mRS 0–1), slight disability (mRS 2), moderate disability (mRS 3), and moderately severe to severe disability (mRS 4–5). Categories with very few participants (mRS 0, *n* = 1; mRS 5, *n* = 2) were merged with their clinically nearest category so that all participants could be retained. Because group sizes were unequal and the variance of RMS scores was not homogeneous across groups (Levene′s test, *p* < 0.001), RMS total scores were compared using the Kruskal–Wallis test, followed by Dunn′s pairwise comparisons with Bonferroni correction. The effect size was expressed as epsilon‐squared (*ε* ^2^). Welch′s ANOVA was performed as a sensitivity analysis. As a secondary, supplementary known‐groups comparison, stroke survivors were also divided into two age groups: young adults (< 60 years) and older adults (≥ 60 years). In addition to the primary mRS‐based known‐groups comparison, we also examined whether the RMS could differentiate between young and older adults with stroke, as age‐related differences in occupational adaptation have been reported in some rehabilitation populations. Given the resulting imbalance in subgroup sizes within our sample, this comparison is presented as a secondary, supplementary analysis. The RMS total scores of these two groups were compared via independent *t*‐test [39]. To evaluate the structural validity of the RMS, an EFA was performed using the principal axis factoring (PAF) method. This method was selected because it does not require the assumption of multivariate normality and is appropriate for ordinal Likert‐type items [40]. Varimax rotation was specified a priori as part of the standard extraction protocol; however, because only a single factor was extracted, rotation had no practical effect on the resulting factor solution.

The internal consistency of the RMS was evaluated using Cronbach′s alpha. A value > 0.7 is deemed acceptable. As a complementary index that does not assume equal item loadings, McDonald′s omega (*ω* *t*) was calculated from the one‐factor model, with 95% confidence intervals obtained by the bootstrap percentile method (5000 replicates of the sample). The same acceptability threshold (> 0.70) was applied. [41]. Item‐level test–retest reliability was assessed using quadratic‐weighted Cohen′s kappa, which is appropriate for ordinal response categories because it gives partial credit to near‐agreements. Linear‐weighted kappa, unweighted kappa, and the percentage of exact agreement are also reported for reference; 95% confidence intervals were estimated using the bootstrap percentile method (5000 resamples). Kappa values > 0.75, 0.40–0.75, and < 0.40 were interpreted as high, moderate‐to‐good, and poor agreement, respectively. [42]. The ICC (2,1) model (two‐way random effects, absolute agreement) was used to calculate relative repeatability, with 95% confidence intervals. The two measurement occasions (test and retest) were treated as a random factor representing possible assessment conditions, consistent with standard practice for test–retest reliability designs even when a single assessor collects both occasions [43]. ICC > 0.8, 0.6–0.8, and < 0.6 indicates high, medium, and low reliability [44]. To calculate absolute repeatability, the SEM was used according to the following formula: $\text{SEM}=\text{SD}\times \sqrt{\left(1\right.}\left.-\text{ICC}\right)$, where SD is the standard deviation of the RMS total score in the full baseline sample (*N* = 100; SD = 6.38). MDC was determined according to the formula: MDC = SEM × 1.96 × √2 [32]; 95% confidence intervals for the SEM and MDC were obtained by substituting the lower and upper limits of the ICC confidence interval into the SEM formula. Confidence intervals for Pearson correlation coefficients were calculated using Fisher′s *z* transformation. Ceiling and floor effects were also investigated, and values < 15*%* were considered acceptable [45].

Baseline characteristics of participants who completed the retest (*n* = 76) and those who did not (*n* = 24) were compared using the Mann–Whitney *U* test for continuous and ordinal variables and Fisher′s exact test (Fisher–Freeman–Halton for variables with more than two categories) for categorical variables. The Mann–Whitney *U* test was chosen because of the small size of the noncompleter group and the nonnormal distribution of some variables.

Statistical calculations for this study were performed using SPSS‐26 software, and the significance level was *p* < 0.05.

## 3. Results

### 3.1. Participants

Table 1 shows the results of the descriptive information of the quantitative and qualitative variables of the study. One hundred people with stroke (52 women) with a mean ± SD age of 71.37 ± 9.68 years were in the range of 50–89 years. The time since stroke was distributed across several categories, ranging from less than 6 months to more than 24 months; 50% of the participants were right hemiplegic, and 84% were right‐handed. The type of stroke of the participants was 93% ischemic and 7% hemorrhagic; 40% of the participants used walking aids. The RMS total scores on the first time (test) were 6.42 ± 2.01, and on the second time (retest), they were 6.37 ± 2.25.

**Table 1:** Demographic and clinical data of the study sample (*N* = 100).

Characteristics		Number (%) and mean ± SD (range)
Age (year)		71.37 ± 9.68 (50–89)
Gender	Male/female	48 (48%)/52 (52%)
Marital status	Single/married	18 (18%)/82 (82%)
Education level	Under diploma/diploma/university degree	73 (73%)/22 (22%)/5 (5%)
Employment status	Employed/retired/jobless	24 (24%)/28 (28%)/48 (48%)
Comorbidities	Hypertension/diabetes/hyperlipidemia/cardiovascular disease/others	36 (36%)/27 (27%)/7 (7%)/25 (25%)/5 (5%)
Living situation	Alone/with spouse or relatives	10 (10%)/90 (90%)
Dominant hand	Right/left	84 (84%)/16 (16%)
Affected side	Right/left	50 (50%)/50 (50%)
Stroke type	Ischemic/hemorrhagic	93 (93%)/7 (7%)
Assistive device use	Yes/no	40 (40%)/60 (60%)
Time since stroke	6/6–12/12–18/18–24 months/more than 24 months	5 (5%)/49 (49%)/28 (28%)/9 (9%)/9 (9%)
Hospital Anxiety and Depression Scale	Anxiety subscale	5.49 ± 2.76 (0–10)
	Depression subscale	6.50 ± 2.51 (0–10)
Canadian Occupational Performance Measurement	Performance	4.92 ± 1.22 (1.75–7)
	Satisfaction	3.34 ± 1.38 (1–6)
Positive Affect Negative Affect Scale	Positive affect	28.91 ± 6.40 (14–45)
	Negative affect	21.09 ± 5.75 (9–34)
Satisfaction With Life Scale		21.80 ± 6.02 (9–34)
Stroke Impact Scale‐16		61.82 ± 15.02 (30–80)
Modified Rankin Scale	No symptoms/no significant disability/slight disability/moderate disability/moderately severe disability/severe disability	1 (1%)/32 (32%)/35 (35%)/15 (15%)/15 (15%)/2 (2%)

### 3.2. Validity

#### 3.2.1. Face and Content Validity

The IS of the items on this scale ranged from 2.80 to 4.14, indicating that the face validity of the questionnaire is at a favorable level. The CVR > 0.80, and the CVI > 0.90 for all items so all items are acceptable and appropriate (Table 2).

**Table 2:** Item‐level content and face validity indices (impact score, content validity ratio, and content validity index) of the Persian version of the Relative Mastery Scale.

Item	Impact score	CVR	CVI
1	3.44	1	1
2	3.96	0.80	1
3	4.14	0.80	1
4	2.80	0.80	0.93
5	3.87	0.80	1
6	2.80	0.80	1

#### 3.2.2. Construct Validity (Hypotheses Testing)

There were moderate correlations between the RMS total score and the COPM‐performance (*r* = 0.63, 95% CI: 0.49–0.73, *p* < 0.001), COPM‐satisfaction (*r* = 0.60, 95% CI: 0.46–0.71, *p* < 0.001), and SWLS (*r* = 0.63, 95% CI: 0.50–0.74, *p* < 0.001). A strong correlation was found between the SIS‐16 and the RMS (*r* = 0.74, 95% CI: 0.63–0.81, *p* < 0.001). Weaker correlations were found with PANAS positive affect (*r* = 0.48, 95% CI: 0.31–0.61, *p* < 0.001) and PANAS negative affect (*r* = −0.40, 95% CI: −0.55 to −0.22, *p* < 0.001) (Table 3). These findings were consistent with our a priori hypotheses: Positive correlations of moderate‐to‐strong magnitude were observed with COPM‐performance, COPM‐satisfaction, SWLS, and SIS‐16, whereas the correlations with PANAS positive and negative affect were comparatively weaker in magnitude, as hypothesized given the more distal relationship between general affect regulation and occupational mastery.

**Table 3:** Correlation between comparator instruments and the Relative Mastery Scale (construct validity/hypotheses testing).

95% CI		RMS‐total *r* (sig)	Variables
COPM	Performance	0.63 (< 0.001)	0.49–0.73
	Satisfaction	0.60 (< 0.001)	0.46–0.71
SWLS		0.63 (< 0.001)	0.50–0.74
SIS‐16		0.74 (< 0.001)	0.63–0.81
PANAS	Positive affect	0.48 (< 0.001)	0.31–0.61
	Negative affect	–0.40 (< 0.001)	−0.55 to −0.22

*Note:* *p* value < 0.05 indicates a statistically significant result.

Abbreviations: COPM, Canadian Occupational Performance Measurement; PANAS, Positive Affect Negative Affect Scale; RMS, Relative Mastery Scale; SIS, Stroke Impact Scale; SWLS, Satisfaction With Life Scale.

#### 3.2.3. Known‐Groups Validity

RMS total scores differed significantly across the four mRS‐based disability groups (Kruskal–Wallis *H*(3) = 51.60, *p* < 0.001, *ε* ^2^ = 0.52). Dunn′s post hoc comparisons with Bonferroni correction showed that the mRS 0–1 group (median 7.0, IQR: 6.0–9.0) scored significantly higher than the mRS 2 (*p* = 0.006), mRS 3 (*p* < 0.001), and mRS 4–5 (*p* < 0.001) groups and that the mRS 2 group scored significantly higher than the mRS 4–5 group (*p* < 0.001). No significant differences were found between mRS 2 and 3 (*p* = 0.79) or between mRS 3 and 4–5 (*p* = 0.15). Welch′s ANOVA yielded the same conclusion (*F*(3, 36.4) = 43.08, *p* < 0.001). As a secondary, supplementary known‐groups comparison, the results did not show a significant difference in the total score of the RMS between the two groups of young adults (< 60 years, *n* = 10, mean ± SD = 5.20 ± 6.46) and older adults (≥ 60 years, *n* = 90, mean ± SD = 2.30 ± 5.96) with stroke (*t* = 1.448, *p* = 0.15). High variability and unequal group sizes may have limited detection of age‐based differences, though a numerical trend favored younger adults (Table 4).

**Table 4:** Known‐groups validity results of the Relative Mastery Scale (*N* = 100).

	**Disability levels**	**Number**	**RMS-total mean ± SD**

Disability level based on modified Rankin scale	mRS 0–1: no symptoms/no significant disability	33	7.15 ± 2.45
	mRS 2: slight disability	35	^∗^3.40 ± 4.13
	mRS 3: moderate disability	15	^∗^0.60 ± 5.37
	mRS 4–5: moderately	17	^∗^^†^−6.18 ± 4.76
	Kruskal–Wallis *H*	51.60	
	*p*	< 0.001	
	Effect size (*ε* ^2^)	0.52	

	**Age groups**	**Number**	**RMS-total mean ± SD**

By age	20–60 years old	10	5.20 ± 6.46
	60 years and older	90	2.30 ± 5.96
	*t*‐test	1.448	
	*p*	0.15	

*Note:* The Kruskal–Wallis test was applied to assess differences among the mRS groups, with *H* reflecting the significance of differences; *t*‐test was used to determine the difference between group means; *p* value < 0.05 indicates a statistically significant result. *t*‐test was used to determine the difference between group means; *p* value < 0.05 indicates a statistically significant result.

Abbreviations: *ε* ^2^, epsilon‐squared; mRS, modified Rankin Scale; RMS, Relative Mastery Scale.

^∗^Significantly different from mRS 0–1.

^†^Significantly different from mRS 2 (Dunn′s post hoc test with Bonferroni correction).

#### 3.2.4. Structural Validity

The appropriateness of the factor‐analysis model and sampling adequacy were evaluated using Bartlett′s test and the Kaiser–Meyer–Olkin (KMO) test. Based on EFA using the PAF method, a single latent factor was extracted with an eigenvalue of 3.54, explaining 65.52% of the total variance. The KMO measure of sampling adequacy was 0.86, and Bartlett′s test of sphericity was significant (*χ* ^2^ = 337.06, *p* < 0.001), indicating the data were suitable for factor analysis. The scree plot also confirmed a single‐factor solution. The extracted factor demonstrated strong loadings for all six items (ranging from 0.67 to 0.87) and extraction communalities ranging from 0.452 to 0.618, indicating a cohesive unidimensional structure consistent with the theoretical framework of the RMS (Table 5) (Figure 1). A parallel analysis was not performed in this study and is noted as a limitation (Table 5) (Figure 1).

**Table 5:** Factor loadings of the Persian Relative Mastery Scale (*N* = 100).

Item	Text	Component
1	Score your performance.	0.82
2	Score your use of resources (e.g., equipment and aids) to achieve the goal.	0.67
3	Measure the level of satisfaction of your performance.	0.87
4	Measure your effort (time‐energy) to achieve the goal.	0.68
5	Measure your achievement.	0.77
6	Measure the level of satisfaction of others, for example, family and friends, of your performance.	0.79

*Note:* KMO = 0.86, Bartlett′s test = 337.06, sig = 0.000.

**Figure 1:**
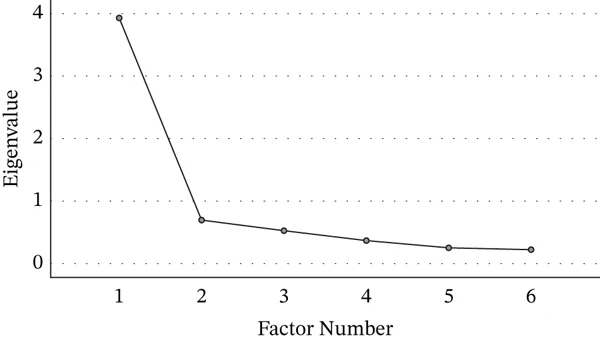
Scree plot of 6‐item Persian Relative Mastery Scale shows 1‐factor with the eigenvalues > 1.

### 3.3. Reliability

#### 3.3.1. Internal Consistency

The Cronbach′s *α* = 0.89 indicates a high level of internal consistency for all six items. If any item were removed, Cronbach′s *α* would range from 0.857 (Item 3) to 0.885 (Items 2 and 4) (Table 6). McDonald′s omega was 0.90 (95% CI: 0.85–0.93), closely matching the alpha estimate. Although factor loadings differed across items (Table 5), the close agreement between the two coefficients indicates that unequal loadings had little effect on the reliability estimate.

**Table 6:** Cronbach′s alpha if item deleted and corrected item‐total correlation for Persian Relative Mastery Scale (*N* = 100).

Relative Mastery Scale (RMS)	Scale mean if item deleted	Scale variance if item deleted	Corrected item‐total correlation	Squared multiple correlation	Cronbach′s alpha if item deleted
1. Score your performance.	2.44	23.804	0.765	0.643	0.866
2. Score your use of resources (e.g., equipment and aids) to achieve the goal.	2.65	28.614	0.634	0.462	0.885
3. Measure the level of satisfaction of your performance.	2.29	24.349	0.810	0.690	0.857
4. Measure your effort (time‐energy) to achieve the goal.	2.03	27.585	0.635	0.478	0.885
5. Measure your achievement.	2.36	25.081	0.722	0.601	0.872
6. Measure the level of satisfaction of others, for example, family and friends, of your performance.	1.53	27.343	0.738	0.589	0.871

#### 3.3.2. Test–Retest Reliability

Item‐level test–retest reliability was assessed after a 14‐day interval (*n* = 76). Quadratic‐weighted kappa coefficients ranged from 0.91 (95% CI: 0.84–0.95; Item 6) to 0.99 (95% CI: 0.97–1.00; Item 4), indicating high reliability for all items (Table 7). Linear‐weighted kappa ranged from 0.82 to 0.97, and unweighted kappa from 0.70 to 0.94. Item 6, which assesses “satisfaction with others′ responses to occupational performance,” showed the lowest agreement (exact agreement 80.3%; unweighted *κ* = 0.70), although 14 of its 15 disagreements were between adjacent response categories, which is reflected in its high weighted kappa (quadratic *κ* = 0.91; linear *κ* = 0.82).

**Table 7:** Item‐level test–retest reliability of the Persian RMS (*n* = 76).

Item	Exact agreement	Unweighted *κ* (95% CI)	Linear‐weighted *κ* (95% CI)	Quadratic‐weighted *κ* (95% CI)
1	92.1%	0.91 (0.82–0.98)	0.96 (0.92–0.99)	0.98 (0.97–1.00)
2	92.1%	0.89 (0.80–0.96)	0.92 (0.85–0.98)	0.95 (0.89–0.99)
3	88.2%	0.84 (0.73–0.93)	0.89 (0.80–0.95)	0.93 (0.86–0.98)
4	96.1%	0.94 (0.85–1.00)	0.97 (0.92–1.00)	0.99 (0.97–1.00)
5	94.7%	0.93 (0.85–0.98)	0.94 (0.87–0.99)	0.96 (0.90–1.00)
6	80.3%	0.70 (0.57–0.82)	0.82 (0.72–0.89)	0.91 (0.84–0.95)

Abbreviations: *κ*, Cohen′s kappa; CI, 95% confidence interval estimated by the bootstrap percentile method (5000 resamples).

Participants who completed the retest (*n* = 76) and those who did not (*n* = 24) did not differ significantly in demographic characteristics, cognitive status, stroke characteristics, or baseline RMS total score (median 4.5 [IQR: −2.0 to 7.0] vs. 4.5 [IQR: 2.0–8.0]; *p* = 0.17) (Table 8). The only exception was COPM‐satisfaction, which was slightly lower among completers (3.20 ± 1.43 vs. 3.80 ± 1.15; *p* = 0.049); this difference did not remain significant after Bonferroni correction for multiple comparisons. Given the small size of the noncompleter group, these comparisons had limited power.

**Table 8:** Comparison of baseline characteristics between participants who completed the retest and those who did not.

Variable	Completed retest (*n* = 76)	Did not complete (*n* = 24)	*p*
Age (year), mean ± SD	71.18 ± 9.86	71.96 ± 9.28	0.83
Gender, male/female, *n* (%)	37 (48.7)/39 (51.3)	11 (45.8)/13 (54.2)	0.82
Marital status, single/married, *n* (%)	17 (22.4)/59 (77.6)	1 (4.2)/23 (95.8)	0.06
Education, under diploma/diploma/university, *n* (%)	55 (72.4)/17 (22.4)/4 (5.3)	18 (75.0)/5 (20.8)/1 (4.2)	1.00
Employment, employed/retired/jobless, *n* (%)	18 (23.7)/22 (28.9)/36 (47.4)	6 (25.0)/6 (25.0)/12 (50.0)	0.95
Living situation, alone/with spouse or relatives, *n* (%)	8 (10.5)/68 (89.5)	2 (8.3)/22 (91.7)	1.00
Dominant hand, right/left, *n* (%)	64 (84.2)/12 (15.8)	20 (83.3)/4 (16.7)	1.00
Affected side, right/left, *n* (%)	40 (52.6)/36 (47.4)	10 (41.7)/14 (58.3)	0.48
Stroke type, ischemic/hemorrhagic, *n* (%)	70 (92.1)/6 (7.9)	23 (95.8)/1 (4.2)	1.00
Assistive device use, yes/no, *n* (%)	33 (43.4)/43 (56.6)	7 (29.2)/17 (70.8)	0.24
Time since stroke, 6/6–12/12–18/18–24/ > 24 months, *n* (%)	3 (3.9)/38 (50.0)/21 (27.6)/7 (9.2)/7 (9.2)	2 (8.3)/11 (45.8)/7 (29.2)/2 (8.3)/2 (8.3)	0.95
MoCA, mean ± SD	21.50 ± 2.19	21.33 ± 1.40	0.47
MMSE, mean ± SD	24.43 ± 2.67	24.21 ± 1.77	1.00
HADS‐anxiety, mean ± SD	5.46 ± 2.87	5.58 ± 2.45	0.88
HADS‐depression, mean ± SD	6.46 ± 2.63	6.62 ± 2.16	0.87
mRS, mean ± SD	2.26 ± 1.20	1.88 ± 0.80	0.24
COPM‐performance, mean ± SD	4.82 ± 1.27	5.26 ± 1.02	0.21
COPM‐satisfaction, mean ± SD	3.20 ± 1.43	3.80 ± 1.15	0.049
SWLS, mean ± SD	21.66 ± 6.50	22.25 ± 4.29	0.74
PANAS‐positive, mean ± SD	28.91 ± 6.79	28.92 ± 5.11	0.97
PANAS‐negative, mean ± SD	20.97 ± 5.98	21.46 ± 5.07	0.77
SIS‐16, mean ± SD	60.34 ± 15.98	66.50 ± 10.40	0.22
RMS total score (baseline), mean ± SD	2.01 ± 6.43	4.42 ± 4.23	0.17

*Note:* Continuous and ordinal variables are presented as mean ± SD and were compared using the Mann–Whitney *U* test; categorical variables are presented as *n* (%) and were compared using Fisher′s exact test (Fisher–Freeman–Halton exact test for more than two categories).

Abbreviations: COPM, Canadian Occupational Performance Measure; HADS, Hospital Anxiety and Depression Scale; MMSE, Mini‐Mental State Examination; MoCA, Montreal Cognitive Assessment; mRS, modified Rankin Scale; PANAS, Positive and Negative Affect Schedule; RMS, Relative Mastery Scale; SIS‐16, Stroke Impact Scale‐16; SWLS, Satisfaction With Life Scale.

#### 3.3.3. Relative Reproducibility

The ICC value for the total score of the RMS was 0.99. Because the ICC > 0.8, the scale demonstrates high relative reliability.

#### 3.3.4. Absolute Reproducibility

The SEM value for the total score of the RMS was 0.638 (95% CI: 0.55–0.88). Given that this value is less than half of the standard deviation and also less than 10% of the total scores, the standard error due to reproducibility is within an acceptable range for the total score.

#### 3.3.5. MDC and Floor and Ceiling Effects

The MDC value was 1.76 (95% CI: 1.53–2.44). The ceiling and floor effects for the total score of the RMS were 3% and 4%, respectively.

## 4. Discussion

To the best of our knowledge, this is the first study investigating the validity and reliability of Part 1 of the RMS in individuals with stroke. The results of this study showed that Part 1 of the Persian version of RMS is a valid and reliable tool for patients with stroke.

### 4.1. Validity

The results of face and content validity showed that all items were acceptable. So, this scale can evaluate the occupational adaptation concept.

Construct validity results showed a moderate correlation (*r* = 0.63) between the COPM‐performance and the RMS total score. The COPM is designed to find out the problem in occupational performance [25]. Regarding relative mastery as one of the key indicators in the OAM [25], this correlation between RMS and COPM reveals that the persons having high relative mastery show better performance during daily activities. This is congruent with occupational adaptation theory, in which greater relative mastery is associated with increasing occupational adaptation abilities. Besides, the “effectiveness” dimension of the RMS, which measures to what extent occupational goals are successfully reached, is highly congruent with COPM, which measures performance in daily activities [12, 25, 46]. Higher COPM scores also represent better efficiency in performing daily activities and are mirrored by the “efficiency” dimension of the RMS, which refers to the optimal use of resources and time [12, 47, 48]. A high correlation (*r* = 0.67) between RMS and COPM scores was also found in the study by Hassani Mehraban et al. [7]. In the present study, the RMS total score showed a moderate correlation with COPM‐satisfaction (*r* = 0.60) and SWLS (*r* = 0.63). COPM‐satisfaction measures performance satisfaction, and SWLS measures life satisfaction [25, 26]. This correlation suggests that people with higher relative mastery tend to report greater satisfaction with their activities and life in general, which may be related to a stronger sense of control over their lives and decisions [49]. A strong correlation was found between the total scores of the RMS and SIS‐16 (*r* = 0.73). Given that higher SIS‐16 scores indicate a better physical status [34], this suggests that physical status is associated with relative mastery and occupational adaptation in managing the disease challenges. However, this strong correlation should be interpreted with caution, as it may partly reflect shared variance with physical functioning or disability severity rather than pure convergence with the broader construct of occupational adaptation captured by the RMS. Compared with other questionnaires, the higher correlation between RMS and SIS‐16 scores may be because of the RMS focusing more on improvements in physical condition and performance rather than psychological factors in occupational adaptation; this closer conceptual and empirical overlap with physical functioning is a limitation to bear in mind when interpreting the RMS as a broad measure of occupational adaptation. In addition, the results of this study showed a weak correlation (−0.40 < *r* < 0.48) between the total score of the RMS and the PANAS score. Previous evidence indicates that improved performance and success in activities are associated with increased positive emotions [47]. This suggests that greater satisfaction with activity performance among individuals with higher RMS scores is associated with increased positive affect. Similarly, higher RMS scores are associated with fewer difficulties or limitations in activity performance, and correspondingly with fewer negative emotions [50].

The results of known‐groups validity showed a significant difference in the RMS among individuals with stroke who have different levels of disability, with higher disability levels associated with lower relative mastery scores (Kruskal–Wallis, *ε* ^2^ = 0.52), a result confirmed by a sensitivity analysis using Welch′s ANOVA. In addition, the results of this secondary, supplementary known‐groups comparison indicated that the scale did not significantly discriminate between young adults (age < 60 years) and older adults (age ≥ 60 years) with stroke based on age. Although the independent *t*‐test revealed no statistically significant difference in RMS scores between younger (< 60 years) and older (≥ 60 years) stroke survivors, this nonsignificant result should be interpreted cautiously given the marked imbalance in subgroup sizes (*n* = 10 vs. *n* = 90), which reflects the clinical epidemiology of stroke and may have limited statistical power to detect age‐specific differences. This finding may also suggest that occupational adaptation after stroke is more proximally related to variables such as motor and cognitive functioning or coping style than to age alone [51–57], though this interpretation requires confirmation in future studies with more balanced age distributions.

The EFA using PAF revealed a robust unidimensional structure for the RMS in stroke survivors. A single latent factor was extracted, accounting for 65.52% of the total variance, with all six items demonstrating strong loadings above 0.68. The high KMO value (0.858) and the significant Bartlett′s test confirmed the adequacy of the sample and the appropriateness of the factor analysis. These psychometric properties support the internal consistency and theoretical coherence of the scale, consistent with the OAM, which conceptualizes relative mastery as a unified construct comprising effectiveness, efficiency, and satisfaction [10]. This result is in line with the research result of George‐Paschal et al. who also reported a one‐factor solution for the RMS in their initial validation [13]. On the contrary, Hassani Mehraban et al. found two factors in their psychometric study (KMO = 0.61, Bartlett′s test = 50.86) [14]. These differences may be attributed to differences in study groups, sample sizes, or methods of analysis. Caregivers may experience mastery as a multidimensional construct involving emotional, interpersonal, and logistical dimensions, whereas stroke survivors may perceive it as a more unified, function‐focused experience. Moreover, Hassani Mehraban et al. prioritized item reduction to enhance brevity and internal consistency, whereas we retained all six items to preserve theoretical coverage within a clinical rehabilitation sample. The emergence of a unidimensional structure in stroke survivors—contrasting with the two‐factor solution observed in caregivers—may reflect conceptual differences in how mastery is experienced across populations. For stroke survivors, mastery might manifest as an integrated adaptation process focused on reclaiming autonomy over bodily functions and daily roles, potentially leading to the observed high interitem coherence. Conversely, the dual demands faced by caregivers—balancing intrapersonal efficacy with situational management—may naturally partition the construct into distinct dimensions. These variations suggest that the dimensionality of the RMS may be context‐dependent. Although a single‐factor structure offers a parsimonious and practical framework that facilitates straightforward interpretability and scoring in clinical settings, further research remains essential to explore the scale′s structural stability across diverse rehabilitation contexts [55]. Further research using confirmatory factor analysis (CFA) across different groups is warranted to establish factorial invariance and theoretical robustness.

### 4.2. Reliability

The results of the present study indicate a high internal consistency (Cronbach′s *α* = 0.89; McDonald′s omega = 0.90) of the RMS items. This finding indicates good internal consistency among the six items and supports the unidimensional structure identified by the EFA. The close agreement between alpha and omega suggests that this finding was not an artifact of alpha′s assumption of equal item loadings. These are comparable with the outcomes obtained by George‐Paschal et al. (Cronbach′s *α* = 0.94) and Hassani Mehraban et al. (Cronbach′s *α* = 0.85) [13, 14].

There was a challenge in determining the best interval to retest the RMS because the original scale never considered test–retest reliability, and the article for its Persian version was published after sampling. It was decided to provide a 2‐week interval before retesting to avoid the memory carryover effect and keep the participants′ mental and physical condition stable. This amount of time is standard across psychometric research, and it is believed to account for these concerns. To ensure that the patients′ conditions did not change during this period, we carefully selected patients and examined criteria such as stability of physical and mental status during the study period. Also, patients who experienced significant changes during the study, such as recurrent stroke or acute physical problems, were excluded from the final analysis. This method ensured that the test–retest results were not affected by real changes in the patients′ conditions [56, 58].

All items of the Persian RMS showed high test–retest reliability when quantified with weighted kappa (quadratic *κ* = 0.91–0.99; linear *κ* = 0.82–0.97). Item 6 showed lower exact agreement (80.3%; unweighted *κ* = 0.70), but nearly all of its disagreements involved adjacent response categories. This may reflect the item′s focus on others′ perceptions (e.g., family or friends), which could be more context‐sensitive than self‐referent items; however, this interpretation remains speculative. ICC for the Persian version of the RMS showed high relative reproducibility (ICC ≥ 0.99) and was consistent with the findings of Hassani Mehraban et al. (ICC ≥ 0.83) [9]. The ICC for the total score demonstrated excellent relative reliability (ICC = 0.98), indicating a high degree of temporal stability. Nevertheless, this exceptionally high value should be interpreted with caution. In addition to the relatively homogeneous clinical characteristics of the study sample and the standard 14‐day retest interval, ICC estimates are influenced by between‐subject variability, which may also have contributed to the observed value. Overall, these findings support the use of the Persian RMS for repeated measurements in individuals with chronic stroke [44].

Adequate absolute repeatability was also found for the total score of the RMS (SEM = 0.63, < 10% of the scale of the total score) and confirmed the RMS as a stable instrument between sessions, reducing the measure errors in the research and intervention effectively. It reduces measurement errors and provides an effective model of research and intervention [43].

The MDC for the total score was 1.76. This MDC value provides a preliminary threshold for distinguishing true change from measurement error and may support clinical monitoring; however, this does not by itself establish responsiveness or a minimal clinically important difference, which require formal longitudinal evaluation. Specifically, any observed change greater than 1.76 points can be interpreted with 95% confidence as exceeding measurement error, rather than necessarily reflecting a clinically meaningful gain in the patient′s adaptive capacity; establishing the clinical meaningfulness of such change requires further responsiveness testing in a longitudinal intervention design. Furthermore, no ceiling or floor effects were found for the RMS.

## 5. Conclusion

According to the results obtained in our research, Part 1 of the Persian version of the RMS has acceptable validity and reliability for examining occupational adaptation in people with stroke and can be used by occupational therapists and rehabilitation researchers in Iran as an effective tool for identifying patients′ problems in the area of occupational adaptation. This scale can help therapists and researchers better identify and evaluate the specific needs of people with stroke, enabling the design and implementation of more appropriate interventions to facilitate the assessment of occupational adaptation and support individualized rehabilitation planning.

## 6. Study Limitations

This study has several limitations that should be considered when interpreting the findings. First, participants were recruited using a nonprobability convenience sampling method, which may limit the generalizability of the findings. Although the sample size met commonly recommended criteria for EFA, larger independent samples are needed to further evaluate the stability and generalizability of the identified factor structure.

Second, individuals with aphasia, cognitive impairment, or severe depression were intentionally excluded to ensure valid self‐report and accurate completion of the RMS and comparator instruments. Although this methodological decision strengthened the internal validity of the psychometric estimates, the findings may not be generalizable to stroke survivors with more severe cognitive, communication, or emotional impairments. Future studies should validate the Persian RMS in these populations as well as in individuals at different stages of stroke recovery.

Furthermore, only Part 1 of the RMS, which consists of structured quantitative items, was evaluated in the present study. Part 2, which includes open‐ended questions designed to explore qualitative aspects of mastery, was not examined in order to maintain consistency with the quantitative psychometric design. Future qualitative or mixed‐method studies may provide a more comprehensive understanding of occupational adaptation by incorporating this component.

In addition, no external gold‐standard instrument is currently available for measuring relative mastery. Therefore, construct validity was evaluated through hypothesis testing using conceptually related outcome measures rather than comparison with a criterion standard. Future studies should continue to examine the construct validity of the Persian RMS in different clinical populations and settings.

Moreover, responsiveness to clinical change was not evaluated in the present study. Although measurement error was assessed using the SEM and the MDC, future longitudinal studies are warranted to determine the ability of the Persian RMS to detect clinically meaningful changes over time and following rehabilitation interventions.

Another limitation is that only EFA was performed to investigate the dimensional structure of the instrument. Future studies should conduct CFA in independent samples to further verify the stability of the identified factor structure.

In addition, 24 participants did not complete the retest assessment; although they did not differ significantly from completers in most baseline characteristics, they showed numerically higher baseline RMS, COPM, and SIS‐16 scores, and the small size of this group limited statistical power. The test–retest estimates should therefore be generalized with caution.

Finally, face and content validity were established using a standard expert‐based approach, including the IS, CVR, and CVI, rather than patient cognitive debriefing interviews. This approach was selected because formal cognitive interviewing requires verbal and executive abilities that may be difficult to standardize in individuals with stroke. Future studies involving stroke survivors with higher cognitive functioning may complement these findings through structured cognitive interviewing.

## Author Contributions

**Mahla Motahari:** Investigation and writing—original draft. **Akram Azad:** Methodology, supervision, and writing—original draft and editing. **Amin Ghaffari:** Formal analysis and writing—review and editing. **Laleh Lajevardi:** Methodology and writing—original draft. **Ghorban Taghizadeh:** Formal analysis.

## Funding

No funding was received for this manuscript.

## Ethics Statement

The study was approved by the Ethics Committee of Iran University of Medical Sciences (Approval Code: IR.IUMS.REC.1402.681; Approval Date: November 14, 2023).

## Consent

Signed informed consent was obtained from all patients.

## Conflicts of Interest

The authors declare no conflicts of interest.

## Supporting information


**Supporting Information** Additional supporting information can be found online in the Supporting Information section. Table S1: COSMIN checklist. Table S2: Overall, COSMIN summary. Table S3: STROBE statement.

## Data Availability

All data used to support the results of this study are included in the article.
