# Supplementary material for: Psychometric Properties of Part 1 of the Persian Relative Mastery Scale in Stroke Survivors
Source: Occup Ther Int. 2026 Nov 15;2026:4334160. doi: 10.1155/oti/4334160 (PMC13770279; doi:10.1155/oti/4334160)
Supplement: Supplementary file 1 [file OTI-2026-4334160-s001.docx]

| **COSMIN checklist for the manuscript is titled:**  Psychometric Properties of Part One of the Persian Relative Mastery Scale in Stroke Survivors | | | | |
| --- | --- | --- | --- | --- |
| **Measurement Property** | **COSMIN Standard** | **Evidence from the Manuscript** | **Methodological Quality** | **Comments / Justification** |
| PROM Development | Development process described | Existing Persian RMS was used; no new item development was performed. | Not Applicable | The study evaluated an existing instrument rather than developing a new PROM. |
| Content Validity | Relevance, comprehensiveness, comprehensibility | 10 occupational therapy experts assessed face validity (Impact Score), CVR and CVI. No cognitive interviews with stroke survivors were performed. | Adequate | Expert evaluation was appropriate; however, COSMIN recommends patient involvement through cognitive interviews whenever feasible. |
| Structural Validity | Dimensionality evaluated appropriately | Exploratory Factor Analysis (PAF), KMO=0.86, Bartlett's test significant, one-factor solution explaining 65.5% variance. Parallel analysis and CFA were not performed. | Adequate | EFA was appropriate, but COSMIN recommends CFA in an independent sample and parallel analysis. |
| Internal Consistency | One-dimensionality established before Cronbach's alpha | One-factor structure confirmed by EFA; Cronbach's α=0.89. | Very Good | Internal consistency was evaluated after assessing structural validity and exceeded the recommended criteria. |
| Reliability (Test–Retest) | Appropriate design and statistical analysis | Two-week interval; ICC (2,1) =0.98 (95% CI 0.98–0.99); item-level Cohen's Kappa=0.70–0.93. | Very Good | Appropriate interval, appropriate ICC model, and excellent reliability estimates. |
| Measurement Error | SEM and MDC reported | SEM=0.638; MDC=1.76. MIC was not determined. | Adequate | Measurement error was quantified, but absence of MIC limits interpretation of meaningful change. |
| Hypothesis Testing (Construct Validity) | A priori hypotheses formulated and tested | Correlations with COPM, SWLS, SIS-16 and PANAS were predefined and consistent with hypotheses. | Very Good | Comparator instruments were theoretically justified and findings supported predefined hypotheses. |
| Known-Groups Validity | Expected differences between known groups | RMS differentiated disability groups based on mRS (p<0.001). Age-group comparison was not significant. | Adequate | Results support known-groups validity; however, the mRS=5 subgroup (n=2) limits robustness. |
| Cross-Cultural Validity / Measurement Invariance | Measurement invariance evaluated | Existing Persian translation was adopted; no DIF or invariance analyses were conducted. | Not Assessed | Cross-cultural validity cannot be confirmed without measurement invariance testing. |
| Criterion Validity | Comparison with gold standard | No gold standard for Relative Mastery exists. | Not Applicable | Criterion validity is not assessable because no accepted reference standard exists. |
| Responsiveness | Ability to detect change over time | Responsiveness was not evaluated. | Not Assessed | Longitudinal studies are required to establish responsiveness. |
| Floor and Ceiling Effects | <15% at minimum or maximum score | Floor effect=4%; Ceiling effect=3%. | Very Good | Both values are below the COSMIN threshold of 15%. |
| Interpretability | Clinical meaning of scores reported | SEM and MDC reported; MIC, normative values and cut-off scores not available. | Adequate | Interpretation is partially supported but remains incomplete without MIC. |

**Overall, COSMIN Summary**

| **Measurement Property** | **Final Rating** |
| --- | --- |
| PROM Development | Not Applicable |
| Content Validity | Adequate |
| Structural Validity | Adequate |
| Internal Consistency | Very Good |
| Reliability | Very Good |
| Measurement Error | Adequate |
| Construct Validity | Very Good |
| Known-Groups Validity | Adequate |
| Cross-Cultural Validity | Not Assessed |
| Criterion Validity | Not Applicable |
| Responsiveness | Not Assessed |
| Floor/Ceiling Effects | Very Good |
| Interpretability | Adequate |

**Overall methodological quality (COSMIN Risk of Bias): *Adequate to Very Good*.**

**STROBE Statement for the manuscript titled:**

Psychometric Properties of Part One of the Persian Relative Mastery Scale in Stroke Survivors

| **Item** | **Recommendation** | **Page(s)** |
| --- | --- | --- |
| **1a** | Study design identified in title/abstract | **Title, Abstract, Methods (pp. 1–2)** |
| **1b** | Informative abstract | **Abstract (p.1)** |
| **2** | Background/Rationale | **Introduction (pp.1–3)** |
| **3** | Objectives and hypotheses | **End of Introduction (p.3)** |
| **4** | Study design | **Methods 2.1 (p.3)** |
| **5** | Setting | **Methods 2.1 (p.3)** |
| **6a** | Participants (eligibility, recruitment) | **Methods 2.1 (pp.3–4)** |
| **7** | Variables | **Methods 2.3–2.5 (pp.4–8)** |
| **8** | Data sources/measurement | **Methods 2.5 (pp.6–8)** |
| **9** | Bias | **Methods 2.1, 2.3 and Study Limitations (pp.3–5, 19–20)** |
| **10** | Study size | **Methods 2.1 (p.3)** |
| **11** | Quantitative variables | **Methods 2.6 (pp.8–10)** |
| **12a** | Statistical methods | **Methods 2.6 (pp.8–10)** |
| **12b** | Subgroup analyses | **Methods 2.6 (p.9)** |
| **12c** | Missing data | **Not reported (NR)** |
| **12d** | Sampling strategy | **Methods 2.1 & 2.6 (pp.3, 8–10)** |
| **12e** | Sensitivity analyses | **Methods 2.1 (post-hoc sensitivity analysis, p.3)** |
| **13a** | Numbers at each stage | **Results 3.1 (pp.10–11)** |
| **13b** | Reasons for non-participation | **Methods 2.1 (p.3)** |
| **13c** | Flow diagram | **Not included (NR)** |
| **14a** | Descriptive characteristics | **Results Table 1 (pp.10–11)** |
| **14b** | Missing data | **Not reported (NR)** |
| **15** | Outcome data | **Results Tables 2–6 (pp.11–15)** |
| **16a** | Main results | **Results 3.2–3.3 (pp.11–15)** |
| **16b** | Category boundaries | **Results Table 4 (p.13)** |
| **16c** | Absolute risk | **Not applicable (NA)** |
| **17** | Other analyses | **Known-groups analysis, factor analysis, reliability analyses (pp.12–15)** |
| **18** | Key results | **Discussion (pp.15–18)** |
| **19** | Limitations | **Study Limitations (pp.19–20)** |
| **20** | Interpretation | **Discussion (pp.15–18)** |
| **21** | Generalisability | **Study Limitations & Conclusion (pp.19–20)** |
| **22** | Funding | **Funding statement (p.21)** |
